# Quality of stapled mesenteric defect closure influences the chance of reopening after laparoscopic Roux-en-Y gastric bypass surgery

**DOI:** 10.1007/s13304-024-01751-4

**Published:** 2024-02-08

**Authors:** F. F. E. Bruinsma, S. J. C. van der Burg, S. El Adel, R. Schouten, S. J. M. Smeets

**Affiliations:** Department of Surgery, Flevoziekenhuis, Hospitaalweg 1, 1315 RA Almere, The Netherlands

**Keywords:** Mesenteric defect, Mesenteric defect closure, Roux-en-Y gastric bypass, Internal hernia

## Abstract

Internal herniation (IH) is a common problem after laparoscopic Roux-en-Y gastric bypass surgery (RYGB). Routine closure of the mesenteric defects (MDs) reduces the risk of IH. Only very few articles report on risk factors for IH or describe detailed closing techniques. There is no consensus yet on the best closing method. The objective of this study is to determine the optimal stapling method for closure of MDs after RYGB. All performed RYGB procedures in our high-volume bariatric institute were included. Quality of the closure was scored in the categories poor, sub-optimal, and optimal, to see if the quality of the closure would predict the chance of reopening of the MDs and, therefore, the chance of IH. During any type of laparoscopy in the follow-up of the patient, the conditions of the MDs were stated, for example during diagnostic laparoscopy in symptomatic patients suspicious for IH or during laparoscopic cholecystectomy. Technically well-executed closure of Petersen’s space (PS) with two rows of staples had a greater chance of still being closed upon re-inspection compared to closure with one row (odds ratio = 8.1; 95% confidence interval [1.2–53.2], *p* = 0.029). Optimal closure of the MD at the jejuno-jejunostomy (JJ-space, JJS) resulted in more closed JJSs upon re-inspection compared to sub-optimal closure (odds ratio = 3.6 [CI 95% 0.8–16.1], *p* = 0.099). Non-optimally closed MDs had higher reopening rates and, therefore, pose an additional risk for IH. Our classification provides a quality assessment of MD closure during RYGB and gives insight into how to optimize surgical technique.

## Introduction

**Fig. 1:**
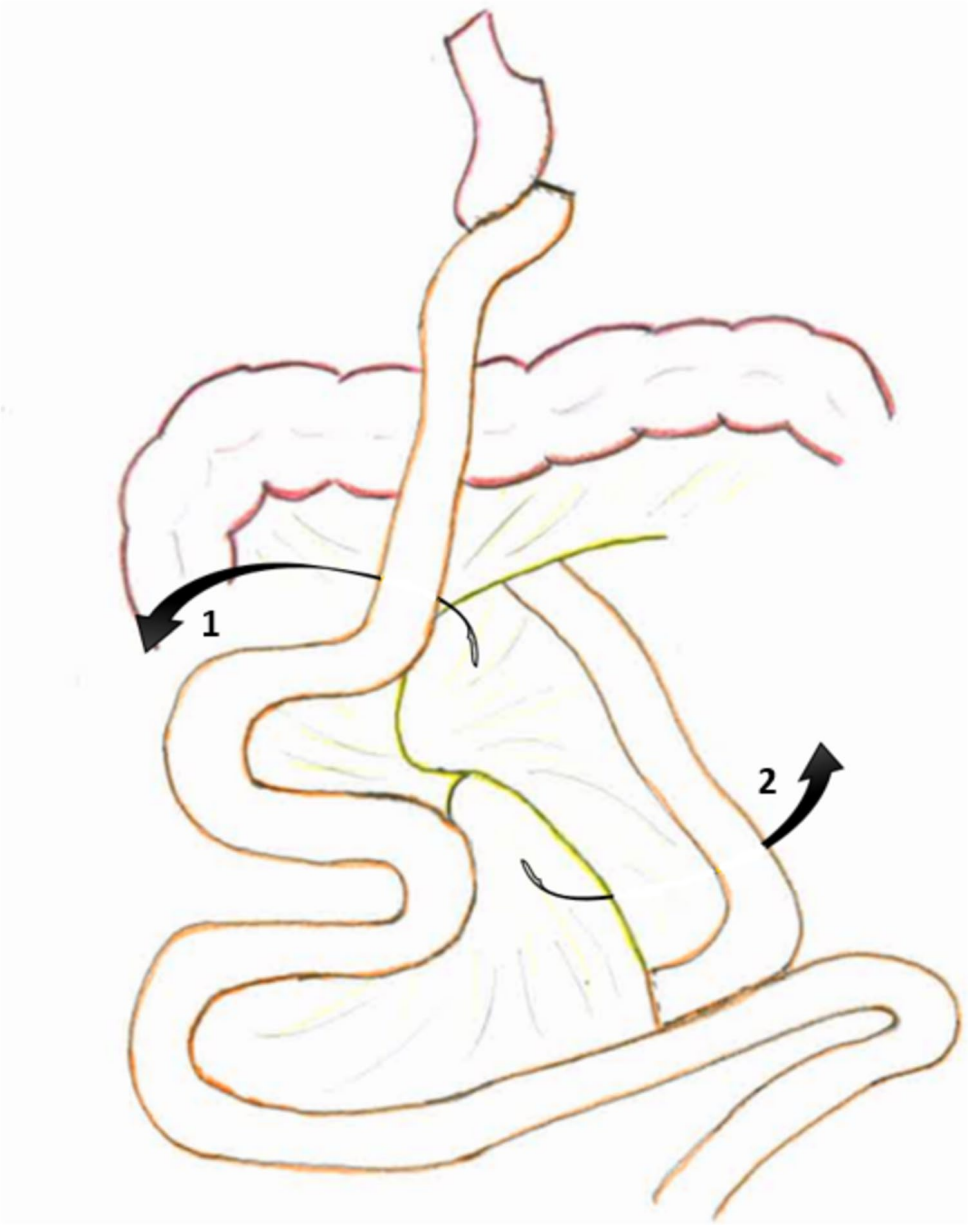
The two hernia sites after Roux-en-Y gastric bypass surgery. 1 = Petersen's space. 2 = jejuno-jejunostomy space

In the past decades, there has been a vast increase in bariatric procedures for the treatment of morbid obesity [1–3]. The second most performed bariatric procedure worldwide between 2012 and 2019 was the Roux-en-Y gastric bypass (RYGB), accounting for almost 300,000 surgeries [1]. RYGB is known for its excellent long-term results with regard to comorbidity resolution and weight loss. Besides the benefits of RYGB, it also creates a risk for complications, with a well-known complication being internal herniation (IH) [4]. During RYGB, two mesenteric defects (MDs) are created where IH may happen [5]. One MD, between the alimentary limb, the transverse mesocolon and the retroperitoneum, is called Petersen’s space (PS); the other MD is at the jejuno-jejunostomy (JJ-space (JJS)), as shown in Fig. 1.

Symptoms of IH can vary from symptoms such as intermittent or post-prandial pain, to persisting pain and acute abdomen [6]. The highest incidence of IH is 1-2 years after surgery, correlating to the time of maximum weight loss. If untreated, IH can result in mesenteric ischemia and eventually mortality [7, 8].

Recent studies have shown that routine closure of the MDs during RYGB reduces the chance of IH [6, 9–16]. A reliable way of closing the MDs is with a stapling device. Aghajani et al. found that closure with staples reduced the chance of IH from 11.7 to 2.5% at 60 months compared to non-closure [17], which was a significant reduction in incidence, but also showed that it did not eliminate IH in all cases, indicating that part of the closed MDs had reopened.

In search for the durability of MD closures, Samur et al. investigated patients with a history of RYGB surgery, all having their MDs closed during primary surgery. If patients went for new intra-abdominal surgery, the status of the MDs was recorded. They found that 52.6% of the patients had at least one MD open or partially open. The cause of open MDs after closure is probably a lack of adhesion formation after surgery which might be influenced by increased traction caused by the rapid loss of mesenteric fat [7]. Samur et al. suggested that the closure of the MDs in two layers might decrease the chance of IH [4].

Currently, there is no consensus about the best practice surgical technique for closing MDs. There is no evidence whether closure of the MDs in two layers influences the chance of reopening of the MDs and eventually IH. This study aims to determine whether closure with two rows of staples results in a smaller chance of reopening.

## Methods

### Study design and informed consent

All patients from 18 years and older who underwent RYGB in our high-volume bariatric institute between April 15, 2019, and February 1, 2022, were prospectively included in a database. First, the criteria for scoring the quality of closure for PS and JJS during RYGB surgery were determined, as described below. Patients with recorded surgery who had their MDs closed with staples were considered eligible for analysis and the quality of their MD closures was assessed. Follow-up of patients happened without any deviation from the local follow-up protocol, meaning that postoperative consultations were at 3 weeks, 3 months, 6 monthly up to 2 years postoperatively, and then yearly afterwards. If patients received any form of upper-abdominal surgery after the primary surgery, the status of their MDs was evaluated, and the findings were categorized into open or closed PS and JJS. Patients were excluded if there was a conversion to laparotomy during the primary surgery or if the closure of PS or JJS was done with materials other than staples. All patients undergoing bariatric surgery in our institute give informed consent to store their data for the purpose of quality control and future research. For this specific study, a waiver for additional informed consent was obtained by the local science committee.

### Surgical procedure

All RYGB procedures were performed with a biliopancreatic limb of 150 cm and an alimentary limb of 75 cm. During surgery, a small gastric pouch is created with a linear stapling device (Covidien, Medtronic®). The integrity of the anastomosis is examined by performing a methylene blue test. An omega loop is created and in an antecolic fashion attached to the pouch with a dorsal linear stapled gastro-jejunostomy. Thereafter, the jejunum is transected and the jejuno-jejunostomy is created via a side-to-side anastomosis. Both MDs are closed using the Multifire Endo Hernia™ of the same manufacturer as the stapling device. The omentum is usually not split.

### Scoring assessment

The quality of closure of PS and JJS was scored by two researchers. Three categories were determined to score the quality of closure of PS: (1) poor closure, (2) sub-optimal closure, and (3) optimal closure.

1. Poor: one single row of staples *OR* a grand space or cavity is left behind (this can be because the deepest point is not included, or because closure started too far from Treitz). If one of these findings is present, score is 1.
2. Sub-optimal: two rows of staples placed parallel and on top of each other *AND* a small space or cavity is left behind *OR* no good identification of transverse mesocolon, meso-ileum and retroperitoneum *OR* closure not along Treitz’s ligament *OR* an incomplete second row of staples. An incomplete second row is defined as covering less than two-thirds of the total distance of the MD (and first staple row). If one of these findings is present, despite the double row of staples, score is 2.
3. Optimal: two rows of staples placed parallel and on top of each other *AND* good identification of transverse mesocolon, meso-ileum and retroperitoneum *AND* closure is along Treitz’s ligament. If all these findings are present, score is 3.

JJS has fewer anatomical variations compared to PS and is considered a less complicated closure. Therefore, two categories were determined for scoring the quality of closure of JJS: (1) sub-optimal, (2) optimal:

1. Sub-optimal: one single row of staples *OR* two rows of staples that do not include the deepest point of the mesenteric folding *OR* a grand space is left behind *OR* an incomplete second row. An incomplete second row is defined as covering less than two-thirds of the total distance of the MD (and first staple row). If one of these findings is present, score is 1.
2. Optimal: two rows of staples placed parallel and on top of each other that include the deepest point of the mesenteric folding. If both findings are present, score is 2.

The space between adjacent staples should not exceed approximately 1 cm. If the space were estimated to be > 1.5 cm, it was considered a small space or cavity and was scored accordingly. To ensure the proper scoring of PS and JJS closure of the analyzed patients, a second researcher scored the video of the primary surgery. These scores were used to determine the inter-observer reliability between the two researchers. If a discordance between the scorings existed, the case was reviewed by the primary investigator (PI, bariatric surgeon).

### Statistical analyses

Statistical analyses were done using SPSS version 26. The primary outcome was dichotomous: ‘mesenteric defect open’ or ‘mesenteric defect closed.’ Variables were ordinal for both JJS and PS. Univariate regression analyses were performed to identify the predictive value of higher quality closure for a closed mesenteric defect during reoperation. Multivariable regression analysis was done to search for confounding variables associated with reopening of the MDs. If the *p*-value was smaller than 0.2, the variable was considered statistically relevant and used in a multivariable regression analysis. A *p*-value < 0.05 was considered statistically significant in all analyses.

The inter-observer reliability was calculated using a Cohen’s kappa coefficient (interpretation of Kappa values ≤ 0 as indicating no agreement; 0.01–0.20 as none to slight; 0.21–0.40 as fair; 0.41– 0.60 as moderate; 0.61–0.80 as substantial; and 0.81–1.00 as almost perfect agreement) [18].

## Results

**Fig. 2:**
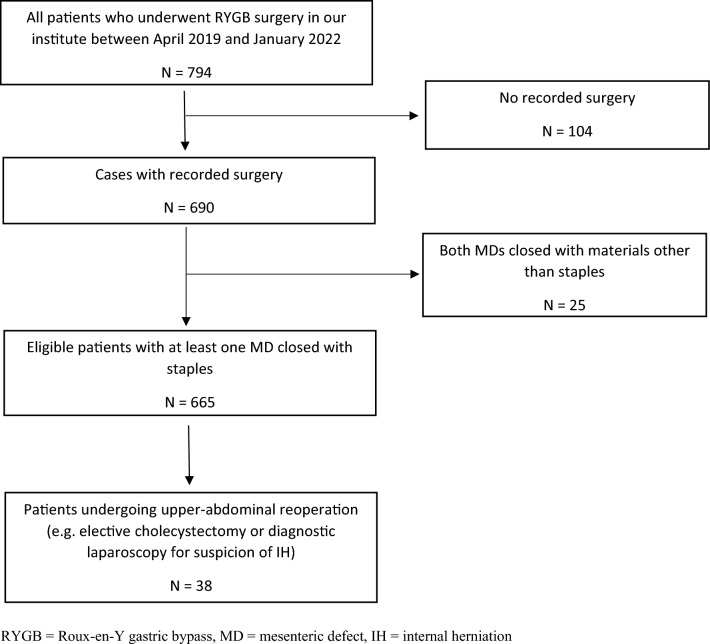
Flowchart of patient inclusion. *RYGB* Roux-en-Y gastric bypass, *MD* mesenteric defect, *IH* internal herniation

Between April 2019 and January 2022, 794 patients underwent RYGB surgery. Of these patients, 690 patients had recorded surgery, of which one patient's JJS closure was not filmed. After exclusion criteria, 665 PSs and 654 JJSs were eligible for analysis. Surgery was performed by three different surgeons, all performing > 100 RYGB per year. Thirty-eight patients (5.7%) underwent a form of reoperation during follow-up and were used for further analysis, see flowchart in Fig. 2. The demographic characteristics of these patients are summarized in Table 1. Reasons for reoperation were cholelithiasis, diagnostic laparoscopy because of suspicion of IH, revision of the gastro-enterostomy, and laparoscopically assisted ERCP (Table 2). The median time between primary surgery and reoperation was 8.5 months (IQR 6.0–14.3). In one patient, the JJS was closed with a V-lock non-absorbable suture and, therefore, only the patient’s PS was assessable for analysis. There were 24 (63.2%) patients with at least one open MD; active IH was seen in 13 patients (34.2%). In 10 out of 37 (27.0%) patients, an open JJS was seen, and an open PS was found in 21 out of 38 (55.3%). In 7 patients, both MDs had reopened.

**Table 1:** Patient characteristics at baseline

	*N* = 38
Age (mean, SD)	39.3 (14.2)
BMI (mean, SD)	40.2 (4.8)
Gender	
Male (*n*, %)	4 (10.5)
Female (*n*, %	34 (89.5)
Comorbidity (*n*, %)	19 (50.1)
Diabetes mellitus type 2 (*n*, %)	4 (10.5)
Hypertension (*n*, %)	9 (23.7)
Osteoarthritis hip or knee (*n*, %)	7 (18.4)
OSAS (*n*, %)	9 (23.7)
Cardiovascular disease (*n*, %)	4 (10.5)
Dyslipidemia (*n*, %)	6 (15.8)
GERD (*n*, %)	9 (23.7)
History of abdominal surgery (*n*, %)	15 (39.5)
Smoking (*n*, %)	7 (18.4)

*n* number, *%* percentage, *SD* standard deviation, *BMI* body mass index, *OSAS* obstructive sleep apnea syndrome, *GERD* gastroesophageal reflux disease

**Table 2:** Type of reoperation

	*N* = 38
Cholecystectomy (*n*, %)	21 (55.3)
DLS due to suspicion of IH (*n*, %)	13 (34.2)
Revision of gastro-enterostomy (*n*, %)	3 (7.9)
Laparoscopically assisted ERCP (*n*, %)	1 (2.6)

*N* number, *%* percentage, *DLS* diagnostic laparoscopy, *IH* internal herniation, *ERCP* endoscopic retrograde cholangiopancreatography

Of the patients with a reopened PS, closure was scored as poor in 9 patients, sub-optimal in 7 and optimal in 5 at primary surgery. Of the patients with closed PS during reoperation, closure was scored as poor in 2 patients, sub-optimal in 6 and optimal in 9. The level of agreement between the two researchers was strong for PS (kappa’s coefficient = 0.849).

A univariate regression analysis comparing the different scores of closure of PS showed that optimal closure (score III) of the mesenteric defect compared with poor closure (score I) is a statistically significant positive predictive value for a closed PS during reoperations (OR = 8.1 [CI 95% 1.2–53.2], *p* = 0.03). Not having a poor closure (score II-III) compared with poor closure also appeared to be a significant positive predictive value for a closed PS during reoperations (OR = 5.625 [CI 95% 1.0–31.1], *p* = 0.048). To identify possible confounders for reopening of the MDs, the following variables were entered in univariate regression analyses: age, sex, T2D, history of previous abdominal surgery, smoking, BMI at screening and percentage BMI loss at the second follow-up moment. It was found that age, sex, T2D, history of abdominal surgery or smoking did not have a statistically relevant influence on the reopening of PS. Percentage BMI loss at the second follow-up moment (mean 4.1 months) did not have a statistically relevant influence on the MDs either. However, univariate regression analysis showed a statistically relevant effect of BMI at screening on the chance of reopening of PS (OR=1.190 [CI 95% 0.986–1.438], p = 0.07). A multivariable analysis with BMI and poor vs. not poor closure showed that BMI is a confounder for the effect of poor vs. not poor closure on the opening of PS since it did not significantly predict reopening anymore (OR= 5.80 [CI 95% 0.9–37.5], *p* = 0.065), as shown in Table 3. Multivariable regression analysis with BMI was not performed with scores 1, 2, and 3, due to the low number of events.

**Table 3:** Univariable and multivariable regression analyses for the influence of the quality of mesenteric defect closure and BMI on not having an open Petersen’s space during reoperation

	Univariable analysis					Multivariable analysis			
Characteristic	*N*	OR	95% CI		*p*	OR	95% CI		*p*
Score	38								
I	11	Ref							
II	13	3.857	0.588	25.292	0.159				
III	14	8.100	1.233	53.200	0.029*				
Score	38								
Poor (I)	11	Ref				Ref			
Not poor (II-III)	25	5.625	1.017	31.097	0.048*	5.798	0.897	37.470	0.065
Score	38								
Not optimal (I-II)	24	Ref							
Optimal (III)	14	3.600	0.902	14.367	0.070				
BMI at screening	38	1.190	0.986	1.438	0.070	1.220	0.968	1.537	0.092

*N* number, *OR* odds ratio, *CI* confidence interval, *BMI* body mass index

*Statistical significance

Out of 10 reopened JJS, 6 were closed sub-optimally, and 4 were closed optimally during primary surgery. The remaining 27 JJS that did not reopen were closed sub-optimally in 8 patients and optimally in 19 patients. Thus, 6 out of 14 (42.3%) sub-optimal closed JJS were open during reoperation, while this only was the case in 4 out of 23 (17.4%) optimally closed JJS. For JJS, the level of agreement between the two researchers was strong as well (kappa’s coefficient = 0.855). Univariate regression analysis did not show a statistically significant predictive value of score II compared with score I (OR= 3.6 [CI 95% 0.8 –16.1], *p* = 0.099, Table 4).

**Table 4:** Univariable regression analysis for the influence of the quality of mesenteric defect closure on not having an open JJ-space during reoperation

	Univariable analysis				
Characteristic	*N*	OR	95% CI		*p*
Score	37				
I	14	Ref			
II	23	3.562	0.786	16.142	0.099

*N* number, *OR* odds ratio, *CI* confidence interval

## Discussion

This study aimed to find an effect of closing PS and JJS consistently with two rows of staples on the chance of reopening. A significant beneficial effect of optimal closure of PS versus poor closure was found, which shows that closing PS close to Treitz and in two complete rows of staples reduces the chance of reopening when compared to closure with one row of staples. The statistically significant effect of poor closure versus ‘not poor’ disappeared after correction for the confounder BMI at screening. However, while having a limited amount of cases in the current study, it seems probable that there is a smaller chance of reopening of PS after RYGB when a double row of staples is used.

In this study, some shortcomings have to be acknowledged. First, objectifying the quality of closure is difficult. The definitions used for poor, sub-optimal and optimal are subjective and are open for discussion. One could suggest that quality should be on a continuous scale instead of an ordinal scale because each closure is different from another. However, using an ordinal scale was necessary to categorize and analyze the patients, and we defined the categories as clearly as possible. This way of categorizing the closures has proven to be a reproducible way to objectify the quality of the closure, given the strong inter-observer agreement. A second shortcoming is that we did not differ between MDs being fully open or partially open. Nevertheless, partially open MDs can cause IH as well, and although bowel herniating through partially open MDs might be more susceptible to ischemia, discrimination between both is probably not relevant. Third, a disadvantage of the current study is the small sample size, with only 38 of 665 patients having received a form of reoperation. Although a statistically significant difference between poor and optimal closure for PS was found, the sub-optimal closure did not statistically differ from poor closure. For JJS, no statistically significant difference was found as well. However, the fact that only 17.4% of the optimally closed JJS was reopened versus 42.3% of the sub-optimally closed JJS suggests an important protective factor of optimal closure. Therefore, longer follow-up with more reoperated patients is needed to solidify these results and possibly create more statistically significant differences between the closure groups.

Last, the current study focused on the occurrence of reopening of the MDs rather than the occurrence of IH. Neither did we analyze symptoms of patients or radiological signs of IH on computed tomography (CT). If signs of IH are found on CT, it appears obvious that an open MD is present, and these findings will inevitably result in surgery. However, if a MD reopened without the presence of active herniation, only surgery can conclude whether the MD is open or closed. A systematic review on the diagnostic accuracy of CT in diagnosing IH showed a sensitivity of 82% and a specificity of 85% [19]. This implies that IH may be overlooked on CT and that diagnostic laparoscopy remains the cornerstone of treatment when IH is suspected. Thus, with the collection of all laparoscopic MD inspections we were able to collect as much data as possible to support our hypothesis that the quality of the initial closure is correlated with the chance to reopen.

Despite the mentioned shortcomings, this study already shows a strong indication of the importance of good-quality closure of the MDs. However, a significant amount of optimally closed MDs still reopened, and there could be multiple explanations for this. Since the scar stabilizes within the first few months after surgery, the first explanation could be the rapid loss of mesenteric fat. The increased traction on the staple line caused by the rapid loss of fat could result in reopening of the MD. Our data do not support this explanation since rapid fat loss—measured by the percentage BMI loss at the second follow-up moment—showed no statistically significant effect on reopening of the MDs. The second possible explanation is an early peritoneum tear on the staple line in the first few hours or days after surgery due to traction on the staple line. Third, a possible factor of influence on reopening of the MDs is the lack of micro-bleedings of the mesentery. Blood promotes tissue regeneration and, therefore, would positively affect mesentery adhesion and scar formation. The presence of micro-bleedings during surgery has not been assessed in this study but could be interesting to analyze in future research. Fourth, high preoperative BMI might also influence the chance of reopening. Because of extensive visceral fat, the closing of the MDs may become more challenging due to more difficultly visualized anatomy (e.g., Treitz and retroperitoneum). Although preoperative BMI was identified as a confounder, a direct effect of preoperative BMI on the chance of reopening was not found.

To the best of our knowledge, only two relevant studies have been published that assessed the status of the MDs during reoperation after laparoscopic RYGB. Samur et al. investigated the MDs of 76 patients in a prospective setup and found that 52.6% of their patients had at least one MD partially or totally open [4]. The current study found a comparable incidence of open MDs after primary closure of 63.2%. This difference can be explained by the indication of surgery, with a higher percentage (34.2% versus 25.0%) of reoperations being performed because of suspicion of IH in our cohort, and therefore, the current study had a higher a priori chance to find open MDs.

Lazaridis et al. included 117 patients in their retrospective analysis and found at least one open MD in 48.8% of their patients [20]. Interestingly, a high percentage of 53.7% of their reoperations were performed because of suspicion of IH (preoperative diagnosis of small bowel obstruction or recurrent abdominal pain), and only 29.6% were reoperated because of cholecystolithiasis. The distribution of these percentages could be explained by their longer median follow-up period of 17 months [IQR 10.0–30.5]. This might be due to the fact that during the first year the largest part of weight loss is achieved, with the concomitant risk of gallstone formation, which makes it possible that the distribution of the indication for surgery shifts towards suspicion for IH when follow-up is longer. However, this altered distribution was not found in the study of Samur et al, despite its long median follow-up period of 22.8 months. Furthermore, it is remarkable that Lazaridis et al. found a preoperative BMI less than 40 kg/m^2^ to be a risk factor for the reopening of MDs, while the current study and the study of Samur et al. did not find a statistically significant effect of preoperative BMI.

Both studies of Samur and Lazaridis show that routine closure of the MDs eliminated the risk of IH in approximately 50% of their patients. This finding is comparable with our findings and underlines the importance of the quality of the closure. Optimizing the quality of the closure will diminish the risk of reopening and therefore the chance of IH. However, the relevance of an open MD is questionable since apparently only a small percentage of patients become symptomatic. In the current study, 13 reoperated patients had active IH, in the studies from Samur et al. and Lazaridis et al, this number was 13 and 10, respectively. Which patients become symptomatic is not clear, but alterations in digestive motility could be key [21]. Understanding these mechanisms and being critical of the results of the surgical closure of MDs might help to minimize the lifelong risk of IH.

The current study was the first to conduct video analysis of the primary surgery and to assess the quality of the MD closure, as we suggested that the quality might affect the risk of IH. Strong evidence on how to close the MDs after RYGB is lacking. A well-established way for closure of the MDs is with a continuous suture, but the use of a stapling machine has proven to be a very effective way as well and is therefore gaining ground in the bariatric field, being a frequently used method in northern Europe [8, 15, 22–24]. Nevertheless, knowledge of how to optimally perform a closure with a stapling device, e.g., in a single row or in two rows, is still required. This study is the first to give insight into how the chances of reopening can be decreased. The clinical impact of these results could affect current recommendations regarding the closure of MDs using a stapling device and might contribute to establishing a guideline.

## Conclusion

The current study showed that the risk of reopening of MDs could be minimized by performing closure of the MDs with two rows of staples. It is important to optimize the quality of the closure to make it firm enough to withstand the traction that the staple line will have to endure in the postoperative period. Our recommendation for all bariatric surgeons who use a stapling device for the closure of MDs would be to pay sufficient attention to the quality of the closure and to close the MDs in two layers. For PS specifically, identify the lowest part of both mesenteric planes in relation to the retroperitoneum and Treitz ligament. This will help to prevent reopening of the MDs and to minimize the chance of IH.

## Data Availability

The data is stored locally so that the study can be reproduced. The data is not stored in a publically available data safe.
